# Supplementary material for: KG-COVID-19: A Framework to Produce Customized Knowledge Graphs for COVID-19 Response
Source: Patterns (N Y). 2020 Nov 9;2(1):100155. doi: 10.1016/j.patter.2020.100155 (PMC7649624; doi:10.1016/j.patter.2020.100155)
Supplement: Document S1. Table S1 [file mmc1.pdf]

PATTER, Volume *2*

Supplemental Information

KG-COVID-19: A Framework to Produce Customized Knowledge Graphs for COVID-19 Response

Justin T. Reese, Deepak Unni, Tiffany J. Callahan, Luca Cappelletti, Vida Ravanmehr, Seth Carbon, Kent A. Shefchek, Benjamin M. Good, James P. Balhoff, Tommaso Fontana, Hannah Blau, Nicolas Matentzoglu, Nomi L. Harris, Monica C. Munoz-Torres, Melissa A. Haendel, Peter N. Robinson, Marcin P. Joachimiak, and Christopher J. Mungall

Source	Description	Version currently ingested	File format
SciBite-CORD-19	Occurrence in the COVID-19 literature of various terms of biological interest, including MeSH terms, GO terms, NCBI gene IDs, HPO terms	V1.5	zip, XML
ChEMBL	Antiviral subset of drugs/compounds relevant to COVID-19 as determined by ChEMBL	ChEMBL 27 (Ingested from ChEMBL API)	json
Drug Central	Drug and drug target data	Version dated May 20, 2020	zip, TSV
TTD	Drug and drug target data	Version 7.1.01 (2019.07.14)	txt
PharmGKB	Drug and drug target data	Version dated 2020-09-05	zip, TSV
GO-CAM models	Protein pathway information	Version from 2020-06-19	XML
GO-plus	Gene ontology	2020-09-10 release	json (OBO-json format)
HPO	Human phenotype ontology	2020-08-11 release	json (OBO-json format)
Mondo	Disease ontology	2020-09-14 release	json (OBO-json format)
CHEBI	Chemical ontology	ChEBI release version 191	json (OBO-json format)
UniProt	Gene/protein data and annotations for SARS-CoV-2	Version dated 2020-09-03	gpa, gpi
STRING	protein-protein interaction data	V11	txt
IntAct	protein-protein interaction data (SARS-CoV-2 specific)	2020-09-26 release	XML (miXML format)
zhou_host_proteins	Protein-protein interaction data from "Network-based drug repurposing for novel coronavirus	Ingested from publication	pdf

	2019-nCoV/SARS-CoV-2"(Zhou et al., 2020)		

Supplement Table 1. Summary of data sources currently ingested by KG-COVID-19. For each source, version/date information for the currently ingested data is also shown. An up-to-date list of URLs from which the data can be downloaded is available at: https://github.com/Knowledge-Graph-Hub/kg-covid-19/blob/master/download.yaml
