# Supplementary material for: KG-COVID-19: A Framework to Produce Customized Knowledge Graphs for COVID-19 Response
Source: Patterns (N Y). 2020 Nov 9;2(1):100155. doi: 10.1016/j.patter.2020.100155 (PMC7649624; doi:10.1016/j.patter.2020.100155)
Supplement: Document S2. Article plus Supplemental Information [file mmc2.pdf]

Article

Patterns

KG-COVID-19: A Framework to Produce Customized Knowledge Graphs for COVID-19 Response

Highlights

- KG-COVID-19 is a framework for producing customized COVID-19 knowledge graphs
- Our knowledge graph and framework is free, open-source, and FAIR
- KG-COVID-19 integrates a wide range of COVID-19-related data in an ontology-aware way
- Our KG has been applied to use cases including ML tasks, hypothesis-based querying

Authors

Justin T. Reese, Deepak Unni, Tiffany J. Callahan, ..., Peter N. Robinson, Marcin P. Joachimiak, Christopher J. Mungall

Correspondence

justinreese@lbl.gov

In Brief

An effective response to the COVID-19 pandemic relies on integration of many different types of data available about SARS-CoV-2 and related viruses. KG-COVID-19 is a framework for producing knowledge graphs that can be customized for downstream applications including machine learning tasks, hypothesis-based querying, and browsable user interface to enable researchers to explore COVID-19 data and discover relationships.

Reese et al., 2021, Patterns *2*, 100155
January 8, 2021
https://doi.org/10.1016/j.patter.2020.100155

CellPress

Patterns

CellPress
OPEN ACCESS

Article

KG-COVID-19: A Framework to Produce Customized Knowledge Graphs for COVID-19 Response

Justin T. Reese,[1,9,*] Deepak Unni,[1] Tiffany J. Callahan,[2] Luca Cappelletti,[3] Vida Ravanmehr,[4] Seth Carbon,[1] Kent A. Shefchek,[5] Benjamin M. Good,[1] James P. Balhoff,[6] Tommaso Fontana,[7] Hannah Blau,[4] Nicolas Matentzoglu,[8] Nomi L. Harris,[1] Monica C. Munoz-Torres,[1,5] Melissa A. Haendel,[5] Peter N. Robinson,[4] Marcin P. Joachimiak,[1] and Christopher J. Mungall[1]
[1]Division of Environmental Genomics and Systems Biology, Lawrence Berkeley National Laboratory, Berkeley, CA 94720, USA
[2]Computational Bioscience Program, Department of Pharmacology, University of Colorado Anschutz School of Medicine, Aurora, CO 80045, USA
[3]Department of Computer Science, University of Milano, 20122 Milan, Italy
[4]The Jackson Laboratory for Genomic Medicine, Farmington, CT 06032, USA
[5]Linus Pauling Institute, Environmental and Molecular Toxicology, Oregon State University, Corvallis, OR 97331, USA
[6]Renaissance Computing Institute, University of North Carolina at Chapel Hill, Chapel Hill, NC 27517, USA
[7]Dipartimento di Elettronica, Informazione e Bioingegneria, Politecnico di Milano, 20133 Milan, Italy
[8]Independent Semantic Technology Contractor, London, UK
[9]Lead Contact
*Correspondence: justinreese@lbl.gov
https://doi.org/10.1016/j.patter.2020.100155

THE BIGGER PICTURE An effective response to the COVID-19 pandemic relies on integration of many different types of data available about SARS-CoV-2 and related viruses. KG-COVID-19 is a framework for producing knowledge graphs that can be customized for downstream applications including machine learning tasks, hypothesis-based querying, and browsable user interface to enable researchers to explore COVID-19 data and discover relationships.

1 2 **3** 4 5 **Development/Pre-production:** Data science output has been rolled out/validated across multiple domains/problems

SUMMARY

Integrated, up-to-date data about SARS-CoV-2 and COVID-19 is crucial for the ongoing response to the COVID-19 pandemic by the biomedical research community. While rich biological knowledge exists for SARS-CoV-2 and related viruses (SARS-CoV, MERS-CoV), integrating this knowledge is difficult and time-consuming, since much of it is in siloed databases or in textual format. Furthermore, the data required by the research community vary drastically for different tasks; the optimal data for a machine learning task, for example, is much different from the data used to populate a browsable user interface for clinicians. To address these challenges, we created KG-COVID-19, a flexible framework that ingests and integrates heterogeneous biomedical data to produce knowledge graphs (KGs), and applied it to create a KG for COVID-19 response. This KG framework also can be applied to other problems in which siloed biomedical data must be quickly integrated for different research applications, including future pandemics.

INTRODUCTION

Although most coronaviruses typically cause common-cold symptoms in humans, three betacoronaviruses have emerged in the past few decades that can cause a range of serious manifestations, including pneumonia and death: the severe acute respiratory syndrome (SARS) coronavirus (SARS-CoV-1), the Middle East respiratory syndrome coronavirus (MERS-CoV), and the novel betacoronavirus that emerged in late 2019, subsequently named SARS-CoV-2, the agent of the disease COVID-19.[1] The rapid spread of SARS-CoV-2 has led to a global pandemic.

This is an open access article under the CC BY license (http://creativecommons.org/licenses/by/4.0/).

COVID-19 is a complex disease involving many biological processes and pathways, each of which involves many genes. Initial symptoms of COVID-19 typically include fever, cough, fatigue, anorexia, anosmia, myalgia, and diarrhea. In some patients, severe illness ensues roughly 1 week after the initial onset of symptoms, and can present with rapidly progressive respiratory failure.[2] In addition to the symptoms highlighted, COVID-19 infections can lead to secondary health problems, such as blood clots,[3] tissue necrosis, organ damage, and, in some cases, cardiac failure. Given that the research community is still learning about COVID-19, understanding its symptoms and their underlying pathological mechanisms, which are still being uncovered, is of vital importance.

Many possible treatments for different aspects and stages of COVID-19 are being actively pursued. Evidence suggests that remdesivir (DrugBank: DB14761), a broad-spectrum antiviral medication, can shorten the time to recovery in adults hospitalized with COVID-19 infection and pneumonia (though the effect is not statistically significant)[4] and more recent evidence suggests that dexamethasone (DrugBank:DB01234), a corticosteroid that suppresses inflammation, may reduce mortality in patients with severe COVID-19.[5] However, currently no treatment is available to prevent progression of COVID-19 to severe disease, and our knowledge of the causes and optimal medical management of the symptoms and resulting clinical complications of COVID-19 are limited.

A large amount of biomedical and molecular data are available to aid the massive research effort to address the COVID-19 pandemic. Before the pandemic began, there existed a large amount of biomedical data for coronaviruses other than SARS-CoV-2 (SARS-CoV and MERS-CoV[6] as well as many other pathogenic and non-pathogenic coronaviruses), such as viral genome and transcriptome sequences, viral/host gene interactions, gene function, epidemiological data, and clinical case data. Much of this information is now also available for SARS-CoV-2. In addition, there is also a large amount of data about drugs that may offer a treatment for COVID-19, as well as the protein targets for each drug.

However, researchers are confronted with a number of technical challenges when trying to use existing data to discover actionable knowledge about COVID-19. The data needed to address a given question are typically isolated in different databases and use different identifiers. These data sources are also often stored in different formats, requiring transformation or preprocessing in order to serve the task at hand. For example, to examine the function of proteins targeted by Food and Drug Administration (FDA)-approved antiviral drugs, one must download and integrate drug, drug target, and FDA approval status data from, for example, Drug Central in a custom-made TSV format[7] and functional annotations from, for example, Gene Ontology in GPAD format.[8] Furthermore, many datasets are updated periodically, which requires researchers to re-download and re-process data in order to perform their analysis on the most current data.

To tackle the daunting challenge of bringing together these disparate sources of information and extracting useful knowledge from them, we used knowledge graphs (KGs). KGs are a way to represent and integrate heterogeneous data and their interrelationships. In a KG, discrete entities or pieces of information form distinct nodes interconnected by edges, where both nodes and edges are typed using a hierarchical system such as an ontology.[9]

For example, nodes of type "*protein*" representing individual entities (such as human ACE2 or SARS-CoV-2 Spike) can be interconnected via edges of type "*orthologous to*" or "*interacts with*," and these nodes can be connected with other kinds of nodes representing diseases, drugs, and so on. This kind of representation is amenable to complex queries (e.g., "which drugs target a host protein that interacts with a viral protein?"), and also to graph-based machine learning techniques.

Related Work

There have been a few parallel efforts to construct KGs to integrate COVID-19 data, each integrating different data sources and constructed for different purposes. Several efforts have constructed KGs by ingesting and transforming scientific literature[10] (https://lg-covid-19-hotp.cs.duke.edu/), some with a few additional types of data also included, such as confirmed case and mortality data (https://github.com/covidgraph/); clinical information, drug trial, and sequencing data (https://www.wikidata.org/wiki/Wikidata:WikiProject_COVID-19); drug, drug trial, and genome sequence data (https://ds-covid19.res.ibm.com/); diseases, chemicals, and genes.[11] Other KG efforts ingest a wider array of data, including diseases, genes, proteins and their structural data, drugs, and drug side effects;[12] pathways, proteins, genes, drugs, diseases, anatomic terms, phenotypes, microbiome (https://spoke.ucsf.edu/); genes, proteins, diseases, phenotypes, genome sequences[13] (https://knetminer.com/); and geographic, viral genes, genes, and proteins (https://github.com/sbl-sdsc/coronavirus-knowledge-graph). Several projects have focused specifically on integrating a wide variety of COVID-19 data to create KGs to investigate drug repurposing[14,15,16] (https://github.com/gnn4dr/DRKG). The effort described here is unique in that it allows users to more flexibly remix specific data types from specific data sources (by virtue of its use of the KGX tool), it integrates more tightly with ontologies (Human Phenotype Ontology [HPO], Mondo, and Gene Ontology [GO]) and with downstream machine learning tools (i.e., Embiggen), it offers a more detailed summary of the contents of its KG in a machine readable format, it covers a wider range of input data sources, and it automatically incorporates new and updated data.

Here, we present a comprehensive COVID-19 KG derived from 13 knowledge sources and containing 377,482 nodes and 21,433,063 edges. The KG is freely available for download at https://kg-hub.berkeleybop.io/kg-covid-19/, and the framework to produce the KG is freely available at https://github.com/Knowledge-Graph-Hub/kg-covid-19. The knowledge graph was constructed using modern ontology best practices whereby different data sources were normalized and merged. KG-COVID-19 allows flexible remixing of component subgraphs for users interested in specific areas. We demonstrate several use cases including graph-based machine learning.

RESULTS

The KG-COVID-19 Framework

We created KG-COVID-19 to address the challenge of integrating data for COVID-19 response. KG-COVID-19 is a

CellPress
OPEN ACCESS

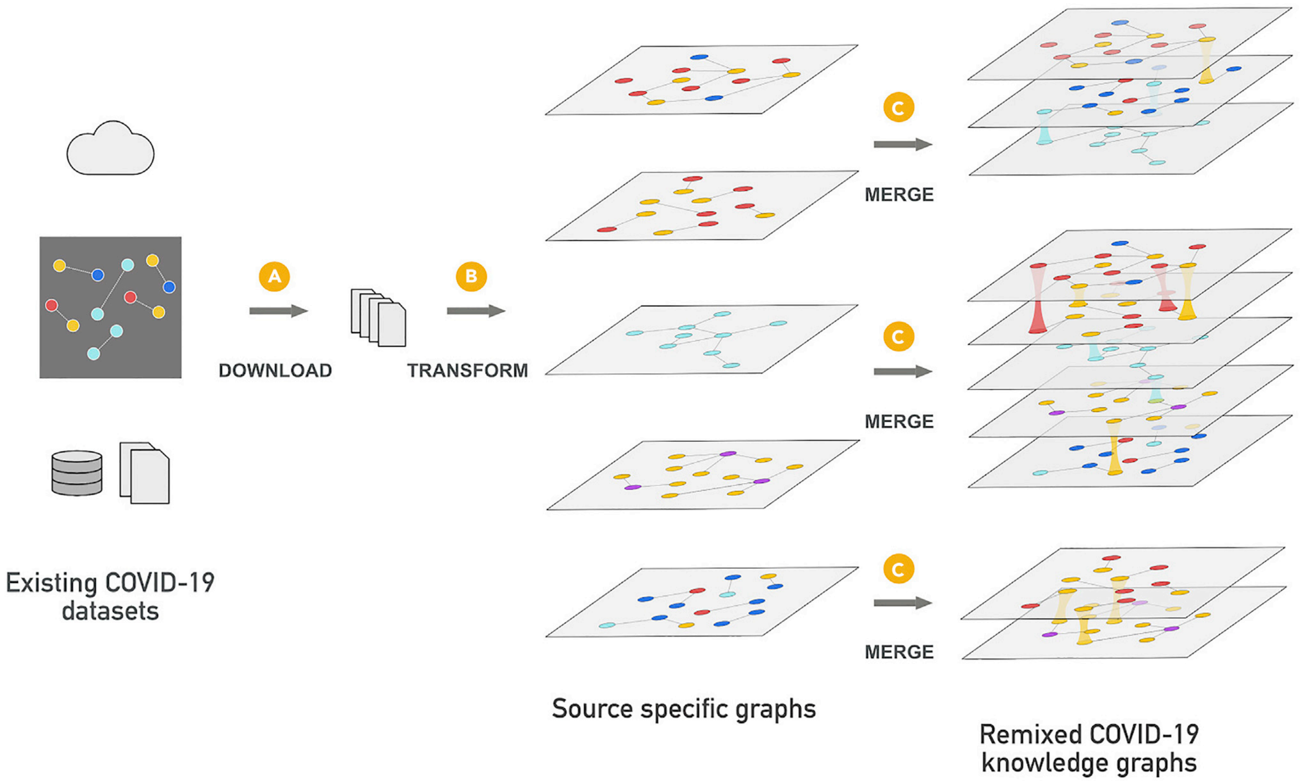

Figure 1. The KG-COVID-19 Framework for Producing KGs

The framework is divided into three modular steps: download, transform, and merge.

(A) The download step retrieves all datasets needed for ingestion using a set of URLs specified in a YAML file.

(B) The transform step applies Python code that is specific to each source to transform the most useful elements of each source and emit a graph in TSV format.

(C) The merge step uses a YAML file to read the user-specified datasets (among those produced in the transform step) and merge them into a single KG. Different YAML files can be constructed to mix and match different input data from B, but each merge operation yields a single merged graph. Both the transform and merge steps rely heavily on KGX, a powerful tool for manipulating knowledge graphs (https://github.com/NCATS-Tangerine/kgx).

framework that enables the creation of customized KGs containing COVID-19 knowledge for different applications. For example, a drug repurposing application would make use of protein data linked with approved drugs, while a biomarker application could use data on gene expression linked with pathways. The methodology is not limited to COVID-19, but could support data integration for any biomedical research effort. In addition, KG-COVID-19 was designed to use a wide variety of human and non-human data resources in order to model important relationships and processes underlying human disease mechanisms. For example, in order to model host response factors in humans, it is necessary to also include mechanisms of virology and viral genes.

Constructing the Knowledge Graph

Our process for generating the KG was designed to support interoperability, preserve provenance, and provide the ability to flexibly mix and match data from different sources. The workflow is divided into three steps: data download (fetch the input data), transform (convert the input data to KGX interchange format), and merge (combine all transformed sources) (Figure 1).

The download step retrieves data from multiple sources using a YAML file that specifies the source URLs (Figure 1A). Our experience has shown that this step is a frequent point of failure in many extract, transform, and load (ETL) pipelines and separating out this step helps mitigate this issue.

The data sources we ingest are focused on our use case: drug repurposing (e.g., drug and drug target data, protein interaction data, ontologies important in disease, such as the HPO and the Mondo disease ontology). However, we also ingest data sources that our user community requests by opening tickets on our project GitHub page (https://github.com/Knowledge-Graph-Hub/kg-covid-19).

The transform step (Figure 1B) involves parsing the input files and transforming them to a graph-based representation. We have devised a simple yet expressive format called KGX interchange format:https://github.com/NCATS-Tangerine/kgx/blob/master/data-preparation.md

a serialization (i.e., the process of converting an object into a format, usually text, that can be re-created when needed) for representing a graph that combines features of Resource Description Framework (RDF) and property graphs (i.e., simplified graphical representations consisting of sets of nodes and edges containing key-value pairs). KGX interchange format consists of two tabular files, one for representing graph nodes and their properties, the other for representing edges and their properties (Figure 2). The format itself is a specialized TSV with certain guidelines on how to represent nodes and edges in a graph.

Original input

DRUG_NAME	STRUCT_ID	TARGET_NAME	TARGET_CLASS	ACCESSION	GENE	SWISSPROT	ACT_VALUE
levobupivacaine	4	Sodium channel protein type 4 subunit alpha	Ion channel	P35499	SCN4A	SCN4A_HUMAN	
levobupivacaine	4	Cytochrome P450 2D6	Enzyme	P10635	CYP2D6	CP2D6_HUMAN	6.706858517
levobupivacaine	4	Potassium voltage-gated channel subfamily H member 2	Ion channel	Q12809	KCNH2	KCNH2_HUMAN	4.89
levobupivacaine	4	Potassium voltage-gated channel subfamily D member 3	Ion channel	Q9UK17	KCND3	KCND3_HUMAN	4.5
levobupivacaine	4	Prostaglandin E2 receptor EP1 subtype	GPCR	P34995	PTGER1	PE2R1_HUMAN	

nodes.tsv

id	name	category	TDL	provided_by
DrugCentral:4	levobupivacaine	biolink:Drug		drug_central
UniProtKB:P35499	SCN4A	biolink:Protein	Tclin	drug_central
UniProtKB:P10635	CYP2D6	biolink:Protein	Tclin	drug_central
UniProtKB:Q12809	KCNH2	biolink:Protein	Tclin	drug_central
UniProtKB:Q9UK17	KCND3	biolink:Protein	Tclin	drug_central

edges.tsv

subject	edge_label	object	relation	provided_by
DrugCentral:4	biolink:molecularly_interacts_with	UniProtKB:P35499	RO:002436	drug_central
DrugCentral:4	biolink:molecularly_interacts_with	UniProtKB:P10635	RO:002436	drug_central
DrugCentral:4	biolink:molecularly_interacts_with	UniProtKB:Q12809	RO:002436	drug_central
DrugCentral:4	biolink:molecularly_interacts_with	UniProtKB:Q9UK17	RO:002436	drug_central

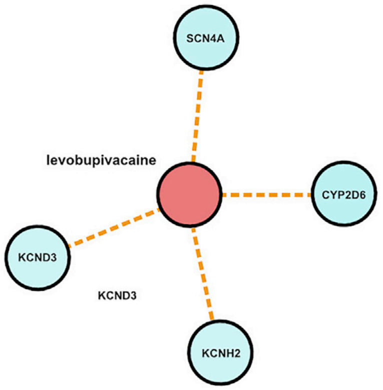

RDF triples

```
<urn:uuid:a5369603-7218-4d4f-8910-5e654103a7e5> <https://w3id.org/biolink/vocab/subject> <http://translator.ncats.nih.gov/DrugCentral_4> .
<urn:uuid:a5369603-7218-4d4f-8910-5e654103a7e5> <https://w3id.org/biolink/vocab/edge_label> <https://w3id.org/biolink/vocab/molecularly_interacts_with> .
<urn:uuid:a5369603-7218-4d4f-8910-5e654103a7e5> <https://w3id.org/biolink/vocab/object> <http://identifiers.org/uniprot/P35499> .
<urn:uuid:a5369603-7218-4d4f-8910-5e654103a7e5> <https://w3id.org/biolink/vocab/relation> <http://purl.obolibrary.org/obo/RO_0002436> .
<urn:uuid:a5369603-7218-4d4f-8910-5e654103a7e5> <https://w3id.org/biolink/vocab/provided_by> "drug_central" .
<urn:uuid:a5369603-7218-4d4f-8910-5e654103a7e5> <http://www.w3.org/1999/02/22-rdf-syntax-ns#type> <https://w3id.org/biolink/vocab/Association> .
<urn:uuid:19093e03-5b76-4e78-b0a6-93e9d3fbd4df> <https://w3id.org/biolink/vocab/subject> <http://translator.ncats.nih.gov/DrugCentral_4> .
<urn:uuid:19093e03-5b76-4e78-b0a6-93e9d3fbd4df> <https://w3id.org/biolink/vocab/edge_label> <https://w3id.org/biolink/vocab/molecularly_interacts_with> .
<urn:uuid:19093e03-5b76-4e78-b0a6-93e9d3fbd4df> <https://w3id.org/biolink/vocab/object> <http://identifiers.org/uniprot/P10635> .
<urn:uuid:19093e03-5b76-4e78-b0a6-93e9d3fbd4df> <https://w3id.org/biolink/vocab/relation> <http://purl.obolibrary.org/obo/RO_0002436> .
<urn:uuid:19093e03-5b76-4e78-b0a6-93e9d3fbd4df> <https://w3id.org/biolink/vocab/provided_by> "drug_central" .
<urn:uuid:19093e03-5b76-4e78-b0a6-93e9d3fbd4df> <http://www.w3.org/1999/02/22-rdf-syntax-ns#type> <https://w3id.org/biolink/vocab/Association>
```

Figure 2. A Typical Transformation of Records from an Input File into Entries in a nodes.tsv and edges.tsv File Representing the Nodes and Edge in a Graph

These nodes and the edge can be further transformed into RDF triples.

Using standards from the Semantic Web, nodes in the graph are identified by Compact Uniform Resource Identifiers (CURIEs).[17] These can be expanded to an Information Resource Identifier (IRI), which is the global identifier for this node. All nodes are assigned a type using the *"category"* node property, and all edges are typed using the *"edge_label"* property. Where possible, one can use classes from the Biolink Model (https://biolink.github.io/biolink-model), a high-level data model for representing biological and biomedical knowledge. Granular typing of nodes is possible by adding additional classes to the *"category"* property. Granular typing of edges is possible by adding a more specific relation to the "*relation"* property. For example, one can use a class from the Relation Ontology (RO) (https://github.com/oborel/obo-relations) to further classify the semantics of an edge.

The merge step (Figure 1C) combines the component datasets into a KG. The merging of two or more graphs is performed by the KGX tool where nodes that have identical CURIEs and edges that have the same source node, edge type, and target node are merged together. When nodes and edges from two different sources do have conflicting properties, we ensure that the properties are preserved. For example, If two nodes had conflicting values for the same property, then we convert the type of the property to a list and keep track of both the values where the order of the list signifies the directionality of the merge. This step is informed by a YAML file that specifies what datasets should be included, to allow for flexible remixing of subgraphs. In addition to selecting different component datasets to be merged, the user can also filter nodes and edges from each source by the node "*category"* and "*edge_label,"* allowing fine-grained control of the resulting graph. By default, all nodes and edges from all component datasets are merged. Optionally, the merged graph can be loaded into any triple/RDF store or Neo4j database.

Design Principles

While our framework offers flexibility in deciding how best to transform each data source, KG-COVID-19 follows some general design principles to maintain the quality of the resulting KG.

Ensure Reproducibility

Our framework is designed to allow users to easily reproduce the KGs used in downstream analysis. The download and transform steps save all ingested data and the transformed data locally after running the pipeline to produce a KG. In addition, we provide prebuilt versions of our KG (https://kg-hub.berkeleybop.io/kg-covid-19/). A new build is constructed each month, and also whenever changes are made to the code in the KG-COVID-19 framework. Each build contains the date the build was constructed, the exact commands that were run to produce the KG, the input data that was ingested, the transformed subgraphs for each source, detailed statistics about the contents of the build, and the KG itself in RDF, KGX TSV, and Blazegraph journal format.

Ensure Interoperability Through Standardized Node and Edge Representations

We use a core set of standardized ontologies and the Biolink Model (https://biolink.github.io/biolink-model), a biological data model for categorizing nodes and edges, to facilitate interoperability and data summarization. To ensure Biolink Model compliance, a Biolink category and a Biolink predicate are required for the categorization of nodes and edges, respectively. Since Biolink predicates are typically very broad in scope, the edge can be further categorized by adding a more specific description in the "*relation*" property using a term from the RO.[18] Categorization using ontologies and the Biolink Model provides a convenient way to assess what types of data have been ingested from each source, record provenance information, and also facilitates interoperability with other transformed datasets.

Ingest Only Relevant Data

Only the subset of features in each dataset that are likely to be useful for downstream applications are preserved, and only statements for authoritative or trusted sources are ingested (for example, assertions about protein interactions are not ingested from a drug database, a trusted resource like the IntAct Molecular Interaction Database would be preferred for protein interactions).

Normalize Identifiers at the Time of Ingest

Identifier (ID) normalization is crucial for ensuring connectedness and the utility of the graph.

We refer to the Biolink Model to provide the preferential order of identifier prefixes to be used for a particular Biolink class. For example, in the case of Gene class (https://biolink.github.io/biolink-model/docs/Gene), the model prescribes HUGO Gene Nomenclature Committee (HGNC), NCBIGene, ENSEMBL, where the order of prefixes matters: identifiers from HGNC namespace are given a higher priority than NCBIGene and ENSEMBL. In the case of Protein class, the model prescribes UniProtKB identifiers. For drugs and other chemical compounds, the model recommends the following: CHEBI, CHEMBL, DrugBank, PubChem. Identifiers can also be normalized by adding cross-references to other identifiers in the "*xrefs*" property of nodes, which is the "*xrefs*" column in the KGX interchange format TSV describing the nodes.

Preserve Provenance

Each ingest adds a "*provided_by*" column in the edge TSV file, which ensures that graphs into which the data are merged (Figure 1C) contain a record of which ingest produced each edge. The preservation of all files used to generate the graph in the download step (Figure 1A) makes it possible to trace each node and edge to the entries in the input file that generated them. PubMed IDs are added to the "*publication*" column, where available, to provide additional provenance.

Downstream Tooling for Querying and Machine Learning

The KG-COVID-19 framework contains tooling for common graph operations. The framework can create training and test datasets in graph form for machine learning applications such as training classifiers or regressors for link prediction (see Experimental Procedures). It also includes a query function that can execute prewritten or custom SPARQL queries on a given SPARQL endpoint (by default, our endpoint: http://kg-hub-rdf.berkeleybop.io/blazegraph/#query).

Current Contents of KG-COVID-19

A schematic diagram of all data sources currently ingested is shown in Figure 3. The Sankey plot in Figure 3 provides insight into the distribution of node and edge types and the connections between nodes in a KG, which is useful for verifying the accuracy of a KG build. The data we ingest are focused on sources relevant to drug repurposing for our downstream querying and machine learning applications, prioritizing drug databases, protein interaction databases, protein function annotations, COVID-19 literature, and related ontologies. The KG contains drug and chemical compound data from several databases, currently DrugCentral,[19] the Pharmacogenomics Knowledgebase (PharmGKB),[20] Therapeutic Target Database (TTD),[21] and ChEMBL;[22] functional annotations and synonyms for coronavirus genes and proteins from the GO; and protein interaction data from STRING[23] and the IntAct Molecular Interaction Database.[24] The IntAct protein interaction dataset contains coronavirus-human protein interaction data taken from 152 COVID-19 publications. We ingest data about COVID-19 scientific publications to identify instances of concepts such as GO terms, UniProt Knowledgebase (UniProtKB) proteins, National Center for Biotechnology Information (NCBI) and HGNC genes, and ChEMBL IDs via SciBite annotations (https://github.com/SciBiteLabs/CORD19) of the COVID-19 Open Research Dataset (CORD-19).[25] To capture ontology-based annotations, the relational graphs for the GO,[8] HPO,[26] and Mondo Disease Ontology[27] are ingested, and annotations are added to the graph as provided by each ingest (https://github.com/Knowledge-Graph-Hub/kg-covid-19/wiki). In addition, we ingest GO Causal Activity Models (GO-CAM) models that capture biological systems such as protein pathways, including those important in SARS-CoV-2 infection.[28]

Use Cases

While we designed KG-COVID-19 to allow flexible reuse and remixing of data to produce custom KGs, our immediate use case is to provide a COVID-19 KG that can be used for machine learning to produce actionable knowledge about COVID-19 (Figure 4). This use case demonstrates several features of KG-COVID-19, namely: normalization and merging of disparate data sources with different namespaces and formats, flexible

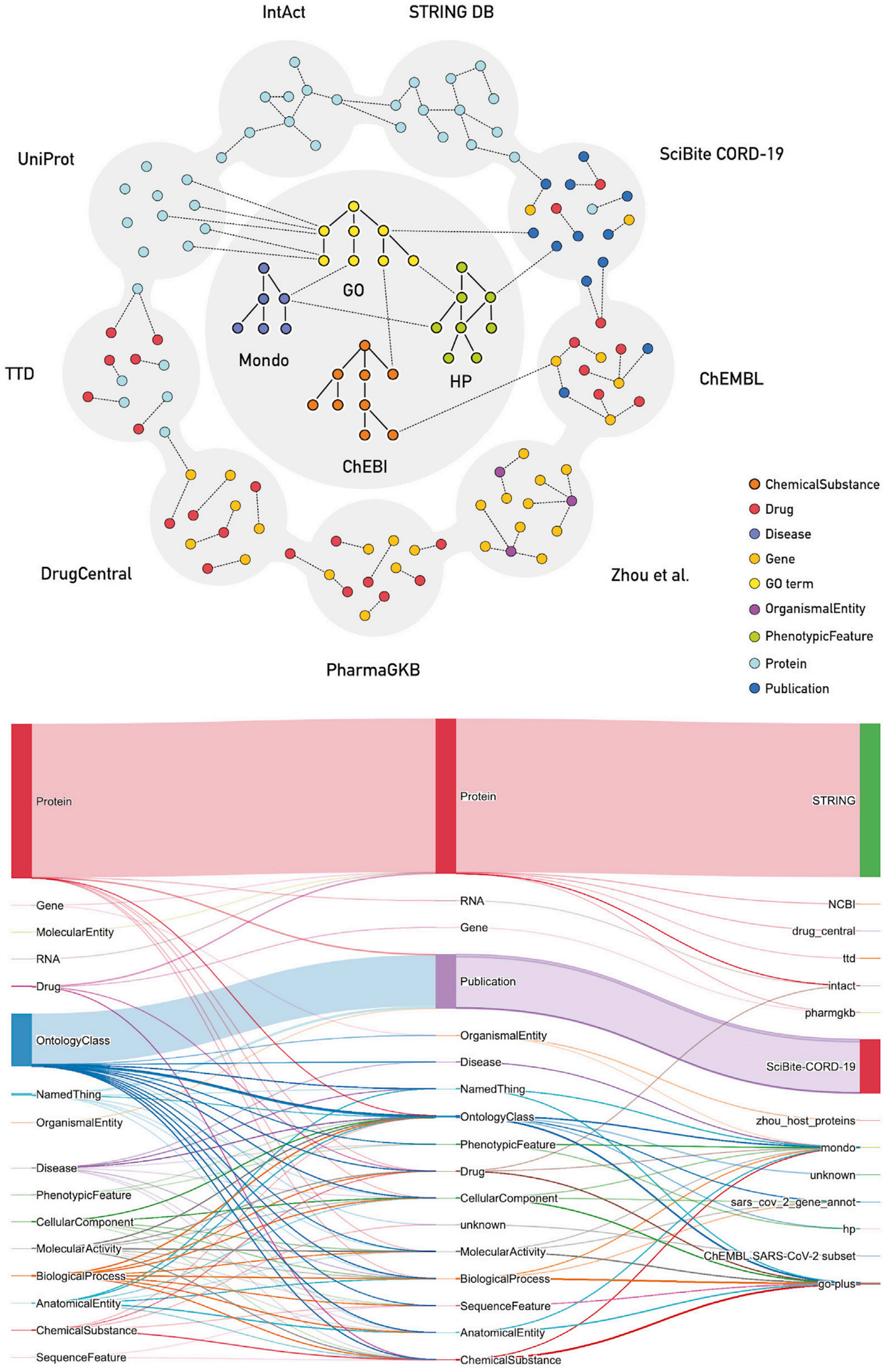

(legend on next page)

CellPress
OPEN ACCESS

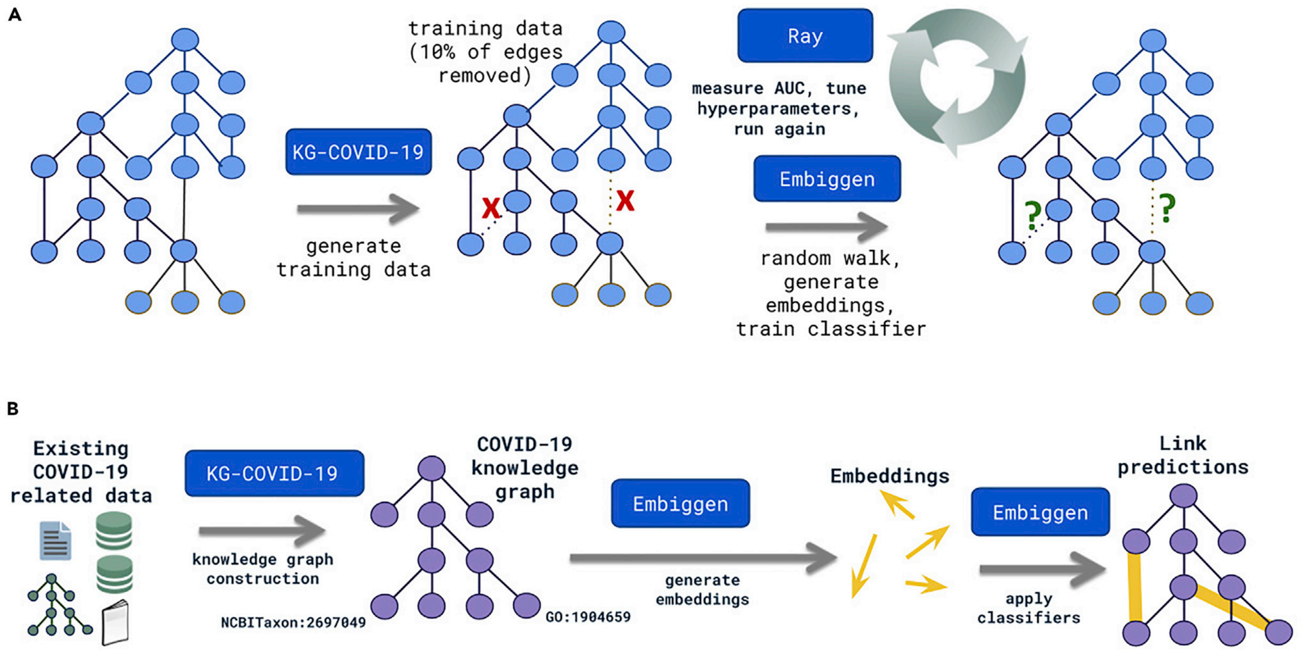

Figure 4. Workflow for Machine Learning Application of KG-COVID-19 Knowledge Graph

(A) In order to train classifiers for use in link prediction, training and test graphs are first produced from the original KG-COVID-19 graph (see Experimental Procedures). These graphs are used by Embiggen to generate random walks, embeddings, and finally a classifier. The test graphs are used to assess the performance of the classifier. This step is performed iteratively in order to identify optimal hyperparameters.

(B) The classifiers are applied to the KG-COVID-19 to perform link prediction in order to identify links that correspond to actionable knowledge: for example, links between drugs and the COVID-19 disease, links between drugs and SARS-CoV-2 protein targets, and links between drugs and host proteins that are involved in COVID-19 disease processes.

remixing of component subgraphs, and a regular update cycle to keep up with new knowledge. We follow the workflow described in Figure 1 to produce the KG-COVID-19 KG. From the final merged graph, KG-COVID-19 produces training and test datasets suitable for machine learning applications (see Experimental Procedures). Embiggen (unpublished data), our implementation of node2vec and related machine learning algorithms, is applied to this KG to generate embeddings, vectors in a low dimensional space which capture the relationships in the KG. Embiggen is trained iteratively to identify optimal node2vec hyperparameters (walk length, number of walks, p, q , and so forth) and to then train classifiers (e.g., logistic regression, random forest, support vector machines) that can be used for link prediction. The trained classifiers can then be applied to produce actionable knowledge: drug to disease links, drug to gene links, and drug to protein links. The latter would indicate a drug that might be useful for COVID-19 treatment.

To demonstrate the usefulness of KG-COVID-19 for machine learning applications, we created embeddings for nodes and edges from the KG-COVID-19 KG and visualized the embeddings in two dimensions using a t-SNE plot (Figure 6). While only the graph structure and no biological typing of nodes was used to generate the embeddings, the nodes of the same type appear to be located closer to each other when projected into latent space than nodes of differing biological types (i.e., genes are closer to other genes than they are to drugs) a phenomenon that is often observed in hierarchically structured data[29] and a feature for which t-SNEs are known.[30] This indicates that the embeddings encode biological information that can be used for machine learning.

While the initial development of KG-COVID-19 has focused on our machine learning applications, other use cases have emerged. As part of the National Virtual Biotechnology Laboratory (NVBL), we have found it useful to perform hypothesis-based querying of the KG to identify viral and human proteins that make attractive drug targets.[31] For example, we have queried the KG to retrieve from our KG host proteins that are known to interact with viral proteins, and these are further filtered according to whether these host proteins are targets of approved drugs (Figure 5). These data are further analyzed with downstream analyses to assess their suitability for drug repurposing. Our KG is also part of a federated query used by the NVBL to collate and share up-to-date information related to COVID-19 and SARS-CoV-2. In addition, the National COVID Cohort Collaborative (N3C) has incorporated our KG as an

Figure 3. Schematic Representation of the Data Currently Ingested into the KG-COVID-19 Knowledge Graph

(Top) Polygons shown correspond to the various data sources currently ingested into the KG, and the small colored circles indicate the data types ingested from this source. (Bottom) Sankey plot showing the Biolink categories for edges in the KG-COVID-19 graph. Left and middle columns show Biolink categories for edges, right column indicates the source of the data from which the edges were derived. Line widths are proportional to the number of edges.

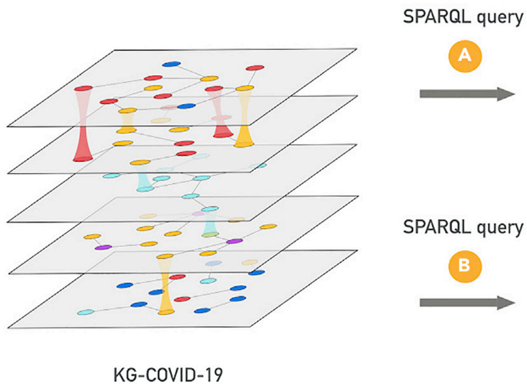

Druggable proteins that interact directly with SARS-CoV-2

SARS-CoV-2 protein	Human Protein	Drug
3CLpro	HDAC2	vironostat
3CLpro	HDAC2	panobinostat
...	...	...

Druggable proteins that interact indirectly with SARS-CoV-2

SARS-CoV-2 protein	Human Protein 1	Human Protein 2	Drug
nsp8	HLA-A	C5	eculizumab
S protein	CCNB1	BCL2	ribavirin
S protein	CCNB1	BCL2	vincristine
...	...	...	...

Figure 5. Hypothesis-Based Querying of KG-COVID-19 Knowledge Graph for Using SPARQL Queries
(Top) An SPARQL query retrieves approved drugs that target human proteins that physically interact with SARS-CoV-2 protein. (Bottom) An SPARQL query retrieves approved drugs that target human proteins that physically interact indirectly with SARS-CoV-2 through another human protein. The suitability of these drugs for repositioning are evaluated by NVBL collaborators, for example by analyzing available structural data to support repositioning.

ontologically informed way to combine their clinical datasets (by virtue of our integration with GO, HPO, and Mondo). The N3C also uses our KG to incorporate all of our transformed and harmonized data, saving them the onerous task of collecting and integrating all of those data sources individually.

DISCUSSION

A "KG-hub" Pattern for Data Sharing

The idea behind a KG-Hub is to provide a platform for building and exchanging knowledge graphs by following a set of guidelines and design principles (https://knowledge-graph-hub.github.io/) that facilitates interoperability and reproducibility. The goal of a KG-Hub is to serve as a collective resource to simplify the process of generating biological and biomedical KGs and thus reducing the barrier for entry to new participants. It also serves as a central resource to enable discovery and exchange of KGs. KG-Hub is designed to be an open-source community-supported resource. We are committed to maintaining this resource and welcome new national and international collaborations to help support this work. Our KG-COVID-19 framework adopts KG-Hub design principles and thus can be considered as the first instance of KG-Hub.

ID Normalization Challenges for SARS-CoV-2 Entities

Since the usefulness of a KG depends on its connectedness, ID normalization is crucial. Normalization of IDs for SARS-CoV-2 entities in particular is challenging, for several reasons. First, SARS-CoV-2 produces identical cleavage products from different polyproteins, and UniProt assigns a different ID to each of these identical cleavage products. For example, UniProt uses PRO_0000338259 to identify the cleavage product nsp5, the 3C-like protease, if it is cleaved from replicase polyprotein 1a, and PRO_0000449623 if it is cleaved from replicase polyprotein 1ab. Protein Ontology, in contrast, uses PR_000050274, irrespective of the polyprotein from which it was cleaved. Note that the UniProt "PRO_" prefix is unrelated to the Protein Ontology namespace. For our KG, it is crucial that identical proteins be represented with a single node such that other information can be efficiently linked to them. We arbitrarily chose PRO_0000449623 as the ID to represent this cleavage product, and all other IDs for this cleavage product are stored as cross-references for this node in our KG. Second, each cleavage product can have a large number of synonyms. For example, nsp5 has at least 40 synonyms that are used in the literature (e.g., 3CL-PRO, 3CLp, Mpro, 3C-like proteinase). Furthermore, some synonyms (e.g., "S" for spike protein) are difficult to recognize when applying NLP to SARS-CoV-2 literature, which represents a further challenge for computationally identifying the occurrences of such entities in text. We have compiled our canonical IDs, synonyms, and cross-references for each SARS-CoV-2 protein and cleavage product in our KG in a publicly available file in GPI format: https://github.com/Knowledge-Graph-Hub/kg-covid-19/blob/master/curated/ORFs/uniprot_sars-cov-2.gpi.

Conclusion

KGs provide a way of integrating heterogeneous data from different sources and combining different data modalities. KG-COVID-19 generates a KG for COVID-19 focused around molecular and chemical information, enabling users to conduct complex queries over relevant biological entities as well as machine learning analyses to generate graph embeddings for making predictions. The lightweight framework we have developed provides a rapid route for bringing together new sources of data and knowledge, including KGs from several different sources, to form a "hub" to support COVID response efforts.

EXPERIMENTAL PROCEDURES

Resource Availability

Lead Contact

Justin Reese, justinreese@lbl.gov, https://orcid.org/0000-0002-2170-2250.

Materials Availability

This study did not generate any physical material.

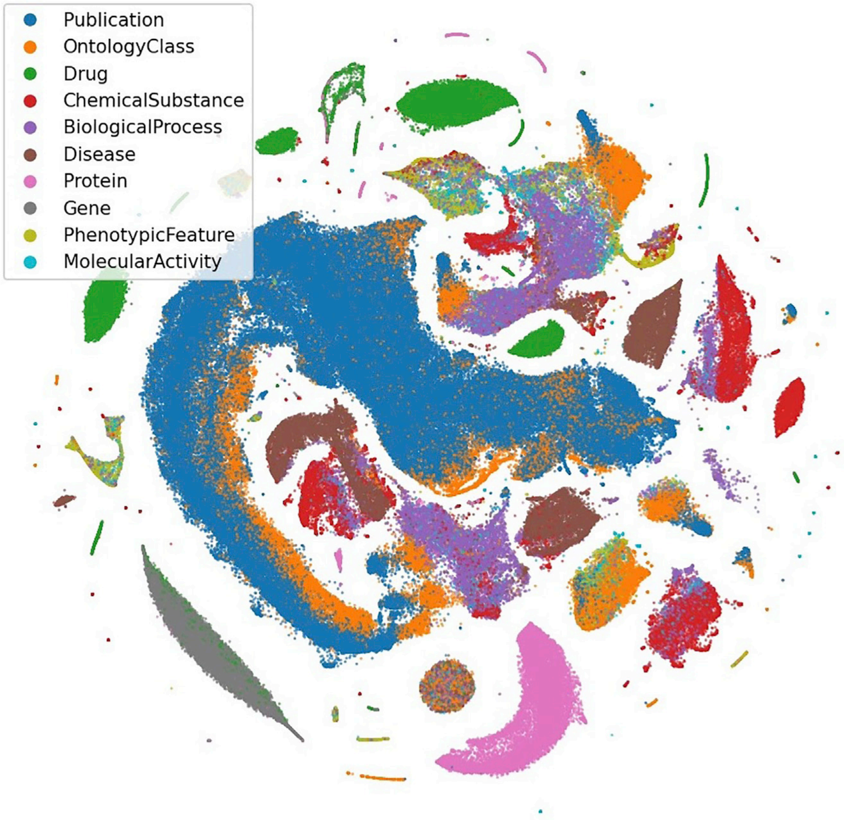

Figure 6. Visualization of KG-COVID-19 Knowledge Graph Node Embeddings Using t-SNE

Embeddings were created for each node in the KG-COVID-19 knowledge graph and t-SNE was performed as described in Experimental Procedures. Nodes categorized with one of the ten most numerous Biolink categories were then selected. Colors indicate the Biolink category for each node.

Data and Code Availability

The Python code for KG-COVID-19 is available from the project wiki: https://github.com/Knowledge-Graph-Hub/kg-covid-19/wiki.https://github.com/Knowledge-Graph-Hub/kg-covid-19/wiki

The Python code is distributed under a BSD3 license.

The KG-COVID-19 KG containing all data sources (in RDF and TSV format) is freely available at: https://kg-hub.berkeleybop.io/kg-covid-19/

An SPARQL endpoint is here:http://kg-hub-rdf.berkeleybop.io/blazegraph/#query.

KG Generation Pipeline

The framework to produce our KG is essentially an ETL system with additional tooling to facilitate downstream uses (e.g., to produce subgraphs for machine learning training, run SPARQL queries). To ensure that the code remains functional and to detect breaking changes in data from upstream sources, we run our pipeline and unit tests regularly using a continuous integration system (https://www.jenkins.io/). This pipeline emits a KG that integrates all available data sources, in both TSV and RDF format, and also loads this KG into a Blazegraph database. A YAML file containing an inventory of the Biolink categories and Biolink associations of all data in the KG is also produced during the merge step (Figure 1). On a commodity server with 200 GB of memory, generation of the KG containing all source data requires a total of 3.7 h (0.13 h, 1.5 h, and 2.1 h for the download, transform, and merge step, respectively), with a peak memory usage of 34.4 GB and disk use of 37 GB.

Generation of Training and Test Edges for Machine Learning Applications

To generate positive edges, a set of positive test edges equal in number to [(1 - train_fraction) * number of edges in input graph] is selected from the edges in the input graph, where train_fraction is a number between 0 and 1 indicating the fraction of the graph to use for training. Positive test edges are selected such that removing them from the graph would not break it into disjoint components. These positive edges are removed from the edges of the input graph and are then emitted as the training edges. A set of negative edges is constructed by randomly selecting pairs of nodes that are not connected by an edge in the input graph. The number of negative edges emitted is equal to the number of positive edges emitted above. If the user requests a validation set, the positive test edges are divided equally to yield positive test and validation sets, and negative test edges are divided equally to yield negative test and validation sets.

Embeddings and t-SNE Plot for Knowledge Graph Visualization

We generated embeddings from our KG using Embiggen, our Python library for graph embedding and machine learning, using node2vec with a skip-gram model, 128 embedding dimensions, and parameters *p* and *q* of 1 (which are typically used default parameters for node2vec).[32] Embiggen is freely available at https://github.com/monarch-initiative/embiggen. These embeddings were used to generate a t-SNE plot that represents the embeddings for each node in two-dimensional space, using MulticoreTSNE (https://github.com/DmitryUlyanov/Multicore-TSNE) (Figure 6).

SUPPLEMENTAL INFORMATION

Supplemental Information can be found online at https://doi.org/10.1016/j.patter.2020.100155.

ACKNOWLEDGMENTS

This work was supported by grants from the Director, Office of Science, Office of Basic Energy Sciences of the U.S. Department of Energy (to J.R., D.U., S.C., N.L.H., M.J., C.J.M.), the Laboratory Directed Research and Development (LDRD) Program of Lawrence Berkeley National Laboratory under U.S. Department of Energy Contract No. DE-AC02-05CH11231, the NIH (Monarch R24 OD011883, Illuminating the Druggable Genome U01 CA239108-01), a Training

Grant from the NLM, NIH to the University of Colorado Anschutz Medical Campus Computational Bioscience Training Program [T15LM009451 to T.J.C.], the National Virtual Biotechnology Laboratory (NVBL), and the Google Cloud COVID-19 Research Grants program.

AUTHOR CONTRIBUTIONS

The KG-COVID-19 framework was conceived and designed by J.R., D.U., M.P.J., C.J.M., T.J.C., N.M., S.C., V.R., and P.N.R.; software was written by J.R., D.U., L.C., T.F., B.M.G., J.P.B., M.P.J., and K.A.S.; and the manuscript was prepared by J.R., D.U., M.P.J., C.J.M., H.B., N.H., M.M.T., and M.A.H.

DECLARATION OF INTERESTS

The authors declare no competing interests.

Received: August 17, 2020
Revised: October 2, 2020
Accepted: November 5, 2020
Published: November 9, 2020

REFERENCES

1. Gandhi, R.T., Lynch, J.B., and Del Rio, C. (2020). Mild or moderate Covid-19. N. Engl. J. Med. *383*, 1757–1766.
2. Berlin, D.A., Gulick, R.M., and Martinez, F.J. (2020). Severe Covid-19. N. Engl. J. Med. Published online May 15, 2020. https://doi.org/10.1056/NEJMcp2009575.
3. Srivastava, K. (2020). Association between COVID-19 and cardiovascular disease. IJC Heart Vasculature *29*, 100583.
4. Beigel, J.H., Tomashek, K.M., Dodd, L.E., Mehta, A.K., Zingman, B.S., Kalil, A.C., et al. (2020). Remdesivir for the treatment of Covid-19. N. Engl. J. Med. https://doi.org/10.1056/NEJMoa2007764.
5. Horby, P., Lim, W.S., Emberson, J., Mafham, M., Bell, J., Linsell, L., et al. (2020). Effect of dexamethasone in hospitalized patients with COVID-19: preliminary report. medRxiv. https://doi.org/10.1101/2020.06.22.20137273.
6. de Wit, E., van Doremalen, N., Falzarano, D., and Munster, V.J. (2016). SARS and MERS: recent insights into emerging coronaviruses. Nat. Rev. Microbiol. *14*, 523–534.
7. Ursu, O., Holmes, J., Bologa, C.G., Yang, J.J., Mathias, S.L., Stathias, V., Nguyen, D.-T., Schürer, S., and Oprea, T. (2019). DrugCentral 2018: an update. Nucleic Acids Res. *47*, D963–D970.
8. The Gene Ontology Consortium (2019). The gene ontology resource: 20 years and still GOing strong. Nucleic Acids Res. *47*, D330–D338.
9. Nickel, M., Murphy, K., Tresp, V., and Gabrilovich, E. (2016). A review of relational machine learning for knowledge graphs. Proc. IEEE *104*, 11–33.
10. Domingo-Fernández, D., Baksi, S., Schultz, B., Gadiya, Y., Karki, R., Raschka, T., et al. (2020). COVID-19 Knowledge Graph: a computable, multi-modal, cause-and-effect knowledge model of COVID-19 pathophysiology. Bioinformatics, In press. https://doi.org/10.1093/bioinformatics/btaa834.
11. Wang, Q., Li, M., Wang, X., Parulian, N., Han, G., Ma, J., et al. (2020). COVID-19 Literature Knowledge Graph Construction and Drug Repurposing Report Generation (bioRXiv), arXiv:2007.00576v3.
12. Khan, J.Y., Khondaker, M.T.I., Hoque, I.T., Al-Absi, H., Rahman, M.S., Alam, T., and Sohel Rahman, M. COVID-19Base: a knowledgebase to explore biomedical entities related to COVID-19. arXiv 2020. arXiv:2005.05954.
13. Hassani-Pak, K., Singh, A., Brandizi, M., Hearnshaw, J., Amberkar, S., Phillips, A.L., Doonan, J.H., and Rawlings, C. (2020). KnetMiner: a comprehensive approach for supporting evidence-based gene discovery and complex trait analysis across species.
14. Ge, Y., Tian, T., Huang, S., Wan, F., Li, J., Li, S., et al. (2020). A data-driven drug repositioning framework discovered a potential therapeutic agent targeting COVID-19. bioRxiv. https://doi.org/10.1101/2020.03.11.986836.
15. Li, X., Yu, J., Zhang, Z., Ren, J., Peluffo, A.E., Zhang, W., et al. (2020). Network Bioinformatics Analysis Provides Insight into Drug Repurposing for COVID-2019. Preprints, 2020030286, https://doi.org/10.20944/preprints202003.0286.v1.
16. Zhou, Y., Hou, Y., Shen, J., Huang, Y., Martin, W., and Cheng, F. (2020). Network-based drug repurposing for novel coronavirus 2019-nCoV/SARS-CoV-2. Cell Discov. *6*, 14.
17. McMurry, J.A., Juty, N., Blomberg, N., Burdett, T., Conlin, T., Conte, N., Courtot, M., Deck, J., Dumontier, M., Fellows, D.K., et al. (2017). Identifiers for the 21st century: how to design, provision, and reuse persistent identifiers to maximize utility and impact of life science data. PLoS Biol. *15*, e2001414.
18. Smith, B., Ceusters, W., Klagges, B., Köhler, J., Kumar, A., Lomax, J., Mungall, C., Neuhaus, F., Rector, A.L., and Rosse, C. (2005). Relations in biomedical ontologies. Genome Biol. *6*, R46.
19. Ursu, O., Holmes, J., Knockel, J., Bologa, C.G., Yang, J.J., Mathias, S.L., Nelson, S.J., and Oprea, T.I. (2017). DrugCentral: online drug compendium. Nucleic Acids Res. *45*, D932–D939.
20. Thorn, C.F., Klein, T.E., and Altman, R.B. (2013). PharmGKB: the Pharmacogenomics knowledge base. Methods Mol. Biol. *1015*, 311–320.
21. Chen, X., Ji, Z.L., and Chen, Y.Z. (2002). TTD: therapeutic target database. Nucleic Acids Res. *30*, 412–415.
22. Gaulton, A., Bellis, L.J., Bento, A.P., Chambers, J., Davies, M., Hersey, A., Light, Y., McGlinchey, S., Michalovich, D., Al-Lazikani, B., et al. (2012). ChEMBL: a large-scale bioactivity database for drug discovery. Nucleic Acids Res. *40*, D1100–D1107.
23. Szklarczyk, D., Gable, A.L., Lyon, D., Junge, A., Wyder, S., Huerta-Cepas, J., Simonovic, M., Doncheva, N.T., Morris, J.H., Bork, P., et al. (2019). STRING v11: protein-protein association networks with increased coverage, supporting functional discovery in genome-wide experimental datasets. Nucleic Acids Res. *47*, D607–D613.
24. Orchard, S., Ammari, M., Aranda, B., Breuza, L., Briganti, L., Broackes-Carter, F., Campbell, N.H., Chavali, G., Chen, C., del-Toro, N., et al. (2014). The MIntAct project–IntAct as a common curation platform for 11 molecular interaction databases. Nucleic Acids Res. *42*, D358–D363.
25. Kohlmeier, S., Lo, K., Wang, L.L., and Yang, J.J. (2020). COVID-19 Open Research Dataset (CORD-19) (Zenodo).
26. Robinson, P.N., Köhler, S., Bauer, S., Seelow, D., Horn, D., and Mundlos, S. (2008). The Human Phenotype Ontology: a tool for annotating and analyzing human hereditary disease. Am. J. Hum. Genet. *83*, 610–615.
27. Mungall, C.J., McMurry, J.A., Köhler, S., Balhoff, J.P., Borromeo, C., Brush, M., Carbon, S., Conlin, T., Dunn, N., Engelstad, M., et al. (2017). The Monarch Initiative: an integrative data and analytic platform connecting phenotypes to genotypes across species. Nucleic Acids Res. *45*, D712–D722.
28. Thomas, P.D., Hill, D.P., Mi, H., Osumi-Sutherland, D., Van Auken, K., Carbon, S., Balhoff, J.P., Albou, L.-P., Good, B., Gaudet, P., et al. (2019). Gene Ontology Causal Activity Modeling (GO-CAM) moves beyond GO annotations to structured descriptions of biological functions and systems. Nat. Genet. *51*, 1429–1433.
29. Kobak, D., and Berens, P. (2019). The art of using t-SNE for single-cell transcriptomics. Nat. Commun. *10*, 5416.
30. van der Maaten, L., and Hinton, G. (2008). Visualizing Data using t-SNE. J. Mach. Learn. Res. *9*, 2579–2605.
31. Office of Science. (2020). National Virtual Biotechnology Laboratory (U.S. DOE Office of Science (SC)).
32. Grover, A., and Leskovec, J. (2016). node2vec: scalable feature learning for networks. KDD *2016*, 855–864.

PATTER, Volume *2*

Supplemental Information

KG-COVID-19: A Framework to Produce Customized Knowledge Graphs for COVID-19 Response

Justin T. Reese, Deepak Unni, Tiffany J. Callahan, Luca Cappelletti, Vida Ravanmehr, Seth Carbon, Kent A. Shefchek, Benjamin M. Good, James P. Balhoff, Tommaso Fontana, Hannah Blau, Nicolas Matentzoglu, Nomi L. Harris, Monica C. Munoz-Torres, Melissa A. Haendel, Peter N. Robinson, Marcin P. Joachimiak, and Christopher J. Mungall

Source	Description	Version currently ingested	File format
SciBite-CORD-19	Occurrence in the COVID-19 literature of various terms of biological interest, including MeSH terms, GO terms, NCBI gene IDs, HPO terms	V1.5	zip, XML
ChEMBL	Antiviral subset of drugs/compounds relevant to COVID-19 as determined by ChEMBL	ChEMBL 27 (Ingested from ChEMBL API)	json
Drug Central	Drug and drug target data	Version dated May 20, 2020	zip, TSV
TTD	Drug and drug target data	Version 7.1.01 (2019.07.14)	txt
PharmGKB	Drug and drug target data	Version dated 2020-09-05	zip, TSV
GO-CAM models	Protein pathway information	Version from 2020-06-19	XML
GO-plus	Gene ontology	2020-09-10 release	json (OBO-json format)
HPO	Human phenotype ontology	2020-08-11 release	json (OBO-json format)
Mondo	Disease ontology	2020-09-14 release	json (OBO-json format)
CHEBI	Chemical ontology	ChEBI release version 191	json (OBO-json format)
UniProt	Gene/protein data and annotations for SARS-CoV-2	Version dated 2020-09-03	gpa, gpi
STRING	protein-protein interaction data	V11	txt
IntAct	protein-protein interaction data (SARS-CoV-2 specific)	2020-09-26 release	XML (miXML format)
zhou_host_proteins	Protein-protein interaction data from "Network-based drug repurposing for novel coronavirus	Ingested from publication	pdf

	2019-nCoV/SARS-CoV-2"(Zhou et al., 2020)		

Supplement Table 1. Summary of data sources currently ingested by KG-COVID-19. For each source, version/date information for the currently ingested data is also shown. An up-to-date list of URLs from which the data can be downloaded is available at:

https://github.com/Knowledge-Graph-Hub/kg-covid-19/blob/master/download.yaml
